# Prevalence and risk factors for efavirenz-based antiretroviral treatment–associated severe vitamin D deficiency: A prospective cohort study

**DOI:** 10.1097/MD.0000000000004631

**Published:** 2016-08-26

**Authors:** Hanna Nylén, Abiy Habtewold, Eyasu Makonnen, Getnet Yimer, Leif Bertilsson, Jürgen Burhenne, Ulf Diczfalusy, Eleni Aklillu

**Affiliations:** a Department of Laboratory Medicine, Division of Clinical Chemistry, Karolinska Institutet at Karolinska University Hospital, Stockholm, Sweden; b Department of Pharmacology, School of Medicine, College of Health Sciences, Addis Ababa University, Addis Ababa, Ethiopia; c Department of Laboratory Medicine, Division of Clinical Pharmacology, Karolinska Institutet, Stockholm, Sweden; d Department of Clinical Pharmacology and Pharmacoepidemiology, University of Heidelberg, Heidelberg, Germany.

**Keywords:** 25-hydroxyvitamin D, cART, CYP2B6, CYP3A4, cytochrome P450, drug metabolism, HIV, TB, tuberculosis, VDR, vitamin D receptor, vitamin D

## Abstract

Initiation of efavirenz-based combination antiretroviral therapy (cART) is associated with Vitamin D deficiency, but the risk factors including efavirenz pharmacokinetics for cART-induced severe vitamin D deficiency (SVDD) and the impact of anti-tuberculosis (TB) cotreatment are not explored. We investigated the prevalence of SVDD in HIV and TB-HIV coinfected patients and associated risk factors for treatment-induced SVDD.

Treatment-naïve Ethiopian HIV patients with (n = 102) or without (n = 89) TB co-infection were enrolled prospectively and received efavirenz-based cART. In TB-HIV coinfected patients, rifampicin-based anti-TB treatment was initiated 4 or 8 weeks before starting cART. Plasma 25-hydroxyvitamin D (25 [OH]D), cholesterol and 4-beta hydroxycholesterol concentrations were measured at baseline, 4^th^, 16^th^, and 48^th^ week of cART. Plasma efavirenz concentrations were determined at 4^th^ and 16^th^ weeks of cART.

TB-HIV patients had significantly lower plasma 25 (OH)D_3_ levels than HIV-only patients at baseline. TB co-infection, low Karnofsky score, high viral load, and high CYP3A activity as measured by plasma 4β-hydroxycholesterol/cholesterol ratios were significant predictors of low 25 (OH)D_3_ levels at baseline. In HIV-only patients, initiation of efavirenz-based cART increased the prevalence of SVVD from 27% at baseline to 76%, 79%, and 43% at 4^th^, 16^th^, and 48^th^ weeks of cART, respectively. The median 25 (OH)D_3_ levels declined from baseline by −40%, −50%, and −14% at 4^th^, 16^th^, and 48^th^ weeks of cART, respectively.

In TB-HIV patients, previous anti-TB therapy had no influence on 25 (OH)D_3_ levels, but the initiation of efavirenz-based cART increased the prevalence of SVDD from 57% at baseline to 70% and 72% at the 4^th^ and 16^th^ weeks of cART, respectively. Median plasma 25 (OH)D_3_ declined from baseline by −17% and −21% at week 4 and 16 of cART, respectively.

Our results indicate low plasma cholesterol, high CYP3A activity, and high plasma efavirenz concentrations as significant predictors of early efavirenz-based cART-induced vitamin D deficiency. Low plasma 25 (OH)D_3_ level at baseline is associated with TB co-infection and HIV diseases progression. Initiation of efavirenz-based cART is associated with high incidence of SVDD, whereas rifampicin based anti-TB therapy co-treatment has no significant effect. Supplementary vitamin D during cART initiation may be beneficial for HIV patients regardless of TB coinfection.

## 1 Introduction

Vitamin D deficiency is associated with many chronic illnesses, including autoimmune, cardiovascular, and infectious diseases like tuberculosis (TB), HIV disease progression, and mortality.^[1–3]^ Plasma 25 (OH)D, the primary circulating form of vitamin D, serves as an indicator of vitamin D status.^[4]^ Vitamin D3 is formed naturally from 7-dehydrocholesterol, a cholesterol precursor, in the skin upon exposure to sunlight. Thus factors influencing 7-dehydrocholesterol levels may result in altered vitamin D and cholesterol levels. Efavirenz-based combination antiretroviral therapy (cART) is associated with changes in both vitamin D^[5–9]^ and cholesterol concentrations,^[10]^ but it is unclear whether there is a correlation between the variation in vitamin D and cholesterol levels during cART and whether this effect is related to drug concentrations.

TB is the most common opportunistic infection and the leading cause of death in people living with HIV. Concomitant HIV and TB treatments are challenging because significant drug–drug interactions, overlapping toxicities, and immune reconstitution inflammatory syndrome.^[11–13]^ Efavirenz and rifampicin, the cornerstones of first-line cART and anti- TB treatments, respectively, are potent inducers CYP2B6 and CYP3A enzymes and transporter proteins.^[10,14,15]^ Efavirenz is mainly metabolized by genetically polymorphic CYP2B6 and to some extent by CYP3A enzymes. Vitamin D is involved in the regulation of CYP3A and CYP2B6 enzyme expression.^[16,17]^ However, CYP3A catalyzes the 4-hydroxylation of 25 (OH)D_3_, and hence CYP3A induction may contribute to drug-induced vitamin D deficiency.^[18,19]^ Long-term CYP2B6 and CYP3A induction by efavirenz is influenced by pharmacogenetic factors relevant for efavirenz disposition.^[10,15,20–22]^ Accordingly, host genetic factors affecting the plasma concentration of CYP3A inducers may also potentially affect the vitamin D levels in patients on long-term treatment with efavirenz or rifampicin. Indeed, a CYP2B6 genotype-based efavirenz dose adjustment was recommended recently to optimize treatment outcomes.^[23,24]^ Implication of inter-individual variations in efavirenz plasma concentration for efavirenz-induced vitamin D deficiency is yet to be investigated.

Previous studies reported that efavirenz-based cART lowers the 25 (OH)D level.^[5–9]^ Poor absorption, lower exposure to the sunshine, and darker skin pigmentation are known risk factors for low 25 (OH)D levels.^[25]^ Data regarding predictors of 25 (OH)D levels during cART are scarce in HIV patients living in tropical areas, where there is an all-year round sunshine. The risk factors for cART-induced severe vitamin D deficiency and the implication of anti-TB co-treatment remain to be investigated, especially in sub-Saharan Africa, where both HIV and TB are major public health problems. In the present study, we hypothesized that high CYP3A induction by rifampicin co-treatment and inter-individual variability in efavirenz disposition may play a role for between-patient variability in vitamin D status during cART. Therefore, the objectives of this study were to identify the prevalence and associated risk factors for low 25 (OH)D levels and severe vitamin D deficiency before starting treatment and during efavirenz-based cART alone or together with rifampicin-based anti-TB co-therapy in HIV or TB-HIV co-infected patients, respectively.

## 2 Materials and methods

### 2.1 Study design and settings

The study design was prospective, comparative, observational, open-label, parallel assignment, 2-arm pharmacogenetic and pharmacokinetic cohort study to identify the prevalence and risk factors for severe vitamin D deficiency at baseline and during efavirenz-based cART with or without rifampicin-based anti-TB therapy. The study was conducted between June 2008 and June 2011 at HIV and TB clinics in Addis Ababa (latitude 9– 1’ N), Ethiopia.

All patients gave written, informed consent to participate in this study. The study protocol received ethics approvals from the Institutional Review Board (IRB) of School of Medicine, Addis Ababa University, National Ethics Review Committee of Ethiopia. The study also received approval from IRB of Karolinska Institutet (Stockholm, Sweden) and was conducted as per International Conference for Harmonization-Good Clinical Practice (ICH-GCP) guidelines.

### 2.2 Study participants

This study was conducted as one of the substudies designed under the umbrella of the broad clinical research project entitled “The HIV-TB Pharmagene Study” in Ethiopia. Details of the main study design, patient enrolment process, and inclusion criteria with follow-up and drug treatments were reported previously.^[26]^ Briefly, for the main study, newly diagnosed HIV-infected (n = 285) and TB-HIV co-infected (n = 208) patients were recruited prospectively and enrolled in parallel and followed up to 48 weeks. The eligibility criteria were ≥18 years of age, not pregnant, and CD4 count ≤200 cells/mm^3^. None of the study participants received isoniazid prophylaxis or other TB treatment for 2 years before enrollment. Treatment adherence was assessed by self-report.

For the present study, a total of 191 patients (102 TB-HIV co-infected patients and 89 HIV only infected patients), with complete set of plasma samples collected at baseline, and at the 4^th^ and 16^th^ weeks of cART, were used to monitor the change in vitamin D during cART. The sample size was calculated for each treatment group considering a moderate effect size, E = 0.3, to detect the change in vitamin D levels before and after efavirenz exposure (paired *t* test) with 80% of study power and α = 5%, the desired sample size was calculated as 87 in each group. Plasma sample collected at 48 weeks of cART from 42 HIV-only patients was also available and used.

### 2.3 Treatment

All HIV patients received cART (600 mg efavirenz-based cART containing either zidovudine/lamivudine/efavirenz or stavudine/lamivudine/efavirenz). Plasma samples for the determination of plasma cholesterol, 4β-hydroxycholesterol, and 25 (OH)D were taken before the initiation of treatment (week 0) and at weeks 4, 16, and 48 of treatment.

**Figure 1:**
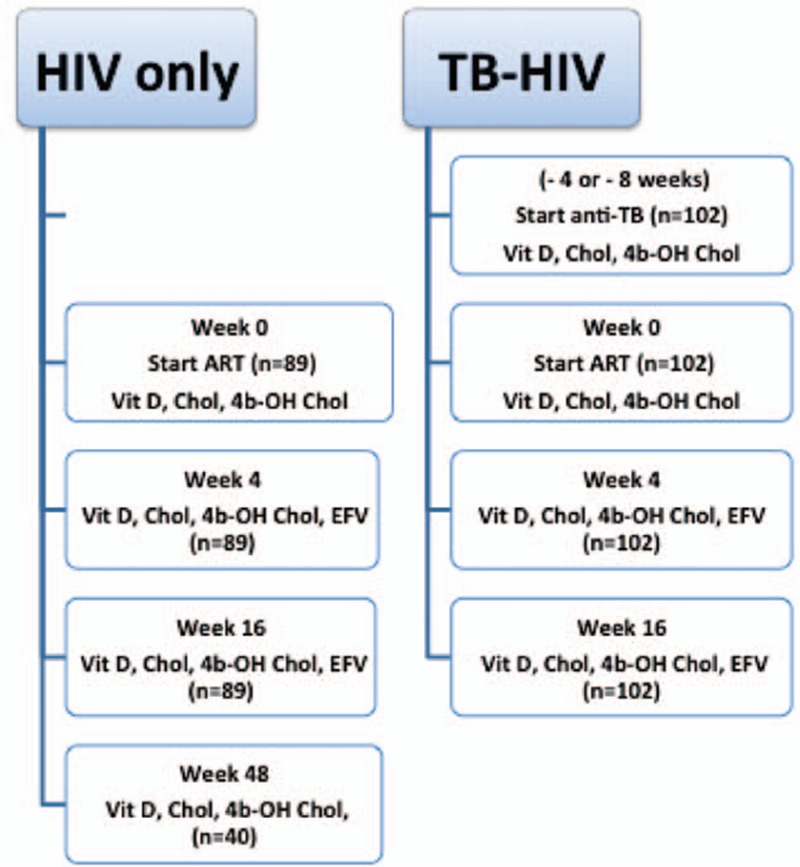
Presentation of the study design, study population, and follow-up period. Study time point for determination of plasma vitamin D (Vit D), cholesterol (Chol), 4β-hydroxylcholesterol (4b-OH chol), and efavirenz (EFV) levels during follow-up period is indicated. HIV-only = HIV patients treated with efavirenz-based cART alone, TB = tuberculosis, TB-HIV = co-infected patients treated with concomitant efavirenz-based cART plus rifampicin-based anti-TB therapy.

All TB-HIV patients (n = 102) initiated rifampicin-based anti-TB treatment 4 (n = 69) or 8 weeks (n = 33) before the initiation of efavirenz-based cART. The short course anti-TB treatment consisted of an initiation phase with rifampicin/isoniazid/pyrazinamide /ethambutol for 2 months followed by a continuation phase with rifampicin/isoniazid for 4 months. Samples for the determination of plasma cholesterol, 4β-hydroxycholesterolm and 25 (OH)D concentrations were collected before starting anti-TB treatment (corresponding to week -4 or -8 weeks of cART), at the initiation of cART (week 0) and weeks 4 and 16 of cART. The study population, follow-up period, and study sampling time points are presented in Figure 1.

Outcome variables were plasma 25 (OH)D level and vitamin D status; vitamin D deficiency (VDD) defined as circulating levels of 25 (OH)D_3_ <20 ng/mL (50 nmol/L), and levels between 21 and 29 ng/mL (51–72.5 nmol/L), considered as insufficient.^[27]^ Severe vitamin D deficiency (SVDD) was defined as plasma 25 (OH)D_3_ level <25 nmol/L.^[25]^ The predicting variables were patient's clinical and laboratory parameters at baseline and during treatment, plasma cholesterol and efvairenz concentrations, and CYP3A enzyme activity as defined by 4β-hydroxylcholesterol/cholesterol ratio.

### 2.4 Quantification of plasma 25 (OH)D concentrations

25 (OH)D_3_ and 25 (OH)D_2_ in plasma were determined by isotope dilution liquid chromatography-tandem mass spectrometry (LC-MS/MS) as described previously.^[28]^ Briefly, 25 (OH)D was extracted from plasma using a Hybrid SPE precipitation 96-well plate (Supelco, Sigma-Aldrich) and isopropanol in acetonitrile (12% v/v, 1% formic acid). 25 (OH) D_3_/D_2_ Plasma Control (level 1) and 25 (OH)D_3_/D_2_ Plasma Calibration Standard were used as quality control and calibrator, respectively (Chromsystems, Munich, Germany). ^2^H_6_–25-hydroxyvitamin D_3_ was used as internal standard (Synthetica AS, Oslo, Norway). Analysis was done on Acquity Quattro Premier LC-MS/MS (Waters, Milford, MA) system equipped with a UPLC BEHC18 column (1.7 μm id, 2.1 × 50 mm, Waters, Milford, MA). The mobile phase was a gradient of 50% to 95% acetonitrile in water (0.1% formic acid). The lower limit of detection (LOD) of 25 (OH)D_3_ was 2.5 nmol/L and the lower limit of quantification (LOQ) 7 nmol/L. The linear range was 2.5 to 625 nmol/L. The relative standard deviation (CV) was 16% (at 33.5 ± 5.4 [n = 39] [mean ± sd]). The LOQ of 25 (OH)D_2_ was 12 nmol/L.

### 2.5 Quantification of plasma cholesterol concentrations

Cholesterol was determined in plasma using a commercial enzymatic method (Cholesterol Chod-PAPP, Roche Diagnostics GMBH, Mannheim, Germany) on a Roche/Hitachi Modular Instrument. The between-day variation was 1.3% at 5 mmol/L.

### 2.6 Quantification of efavirenz plasma concentrations

Plasma samples for determination of efavirenz were collected at weeks 4 and 16 of cART in both treatment groups. Plasma efavirenz concentrations were determined using LC-MS/MS as described previously.^[20,26,29]^ Briefly, protein precipitation was performed using acetonitrile containing internal standards (^13^C_6_-efavirenz and ^2^H_4_–8-hydroxy-efavirenz, respectively). Analysis was performed using a Synergi Fusion RP chromatography column (Phenomenex, Torrance, CA) and mobile phases containing ammonium acetate (5 mmol/L, acidic), methanol, and acetonitrile. The lower limits of quantification in plasma were 10.0 ng/mL. The efavirenz calibration range was 10 to 10000 ng/mL. The method was validated according to the FDA validation guidelines and fulfilled all criteria concerning accuracy, precision, recovery, linearity, and stability.

### 2.7 Statistical analysis

Comparison of median plasma 25 (OH)D_3_ levels between treatment groups was done using the Mann–Whitney test. Plasma 25 (OH)D_3_, cholesterol, efavirenz concentrations, and 4β-hydroxycholesterol/cholesterol ratios were log-transformed (base 10) before applying the *t* test, repeated measure analysis of variance (ANOVA), and regression analysis. Pairwise comparison of data from baseline within and between treatment groups was made using paired and unpaired *t* test, respectively. For each patient, the percent change in plasma 25 (OH)D_3_ level from baseline to the 4^th^, 16^th^ and 48^th^ weeks of cART was calculated using the following formula: 
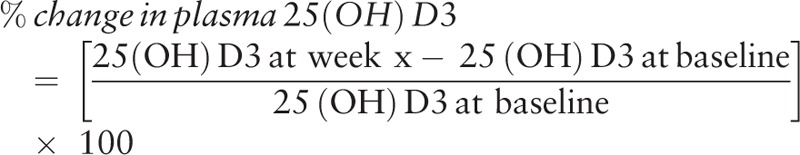


Repeated measure ANOVA was used to analyze the change in log plasma 25 (OH)D_3_ levels over time. Univariate followed by multivariate linear regression analysis was performed to identify predictors of low plasma 25 (OH)D_3_ levels at baseline and during treatment. Predictor variables that resulted in a *P* value <0.1 in the univariate regression analysis was entered into a backward stepwise multivariate regression analysis to identify significant predictors in the final model. Likewise logistic regression was done to determine predictors of severe vitamin D deficiency before and during cART. Statistical analyses were performed using Statistica version 12 (StatSoftInc, Tulsa, OK) and SPSS Statistics (IBM Corporation, Somers, NY) software, version 23.0. GraphPad Prism version 5.0 for Windows (Graph Pad, La Jolla, CA) was used for graphical presentations. A *P* value <0.05 was considered significant.

## 3 Results

The baseline sociodemographic, clinical, and laboratory characteristics of study participants stratified by treatment group are presented in Table 1. The best measure of vitamin D status is the concentration of 25 (OH)D_3_ + 25 (OH)D_2_ in plasma. Very few patients had 25 (OH)D_2_ levels above the lower LOQ; hence, all statistical calculations were done using only plasma 25 (OH)D_3_ data.

**Table 1:**
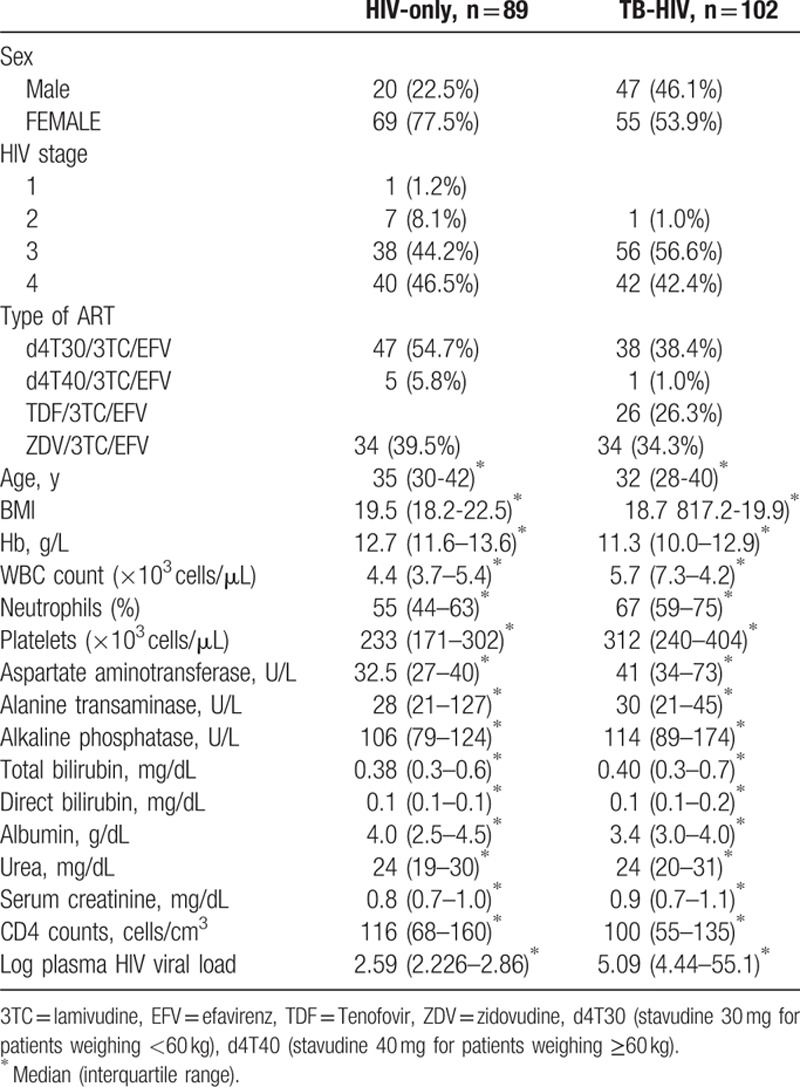
Baseline demographic, clinical, and laboratory characteristics of the study participants.

### 3.1 Plasma 25 (OH)D3 levels at baseline and during treatment

Comparisons of the median plasma 25 (OH)D_3_ levels at baseline and during cART and the median percent change in plasma 25 (OH)D_3_ levels during therapy between the 2 treatment groups are presented in Table 2. The pretreatment median plasma 25 (OH)D_3_ concentration was significantly lower in TB-HIV coinfected patients than in HIV patients without TB. In the HIV-only cohort, initiation of efavirenz-based cART dramatically lowered the median plasma 25 (OH)D_3_ levels by −40%, −50%, −14% at week 4, 16, and 48 of cART, respectively (Table 2). Although in TB-HIV co-infected patients, previous treatment with rifampicin-based anti-TB drugs for 4 or 8 weeks resulted in no significant change in plasma 25 (OH)D_3_ from baseline. However, initiation of efavirenz-based cART co-treatment led to a gradual decline in plasma 25 (OH)D_3_ level and the median percent change of 25 (OH)D_3_ from baseline by 4^th^ and 16^th^ of weeks of cART was −17% and −21%, respectively.

**Table 2:**
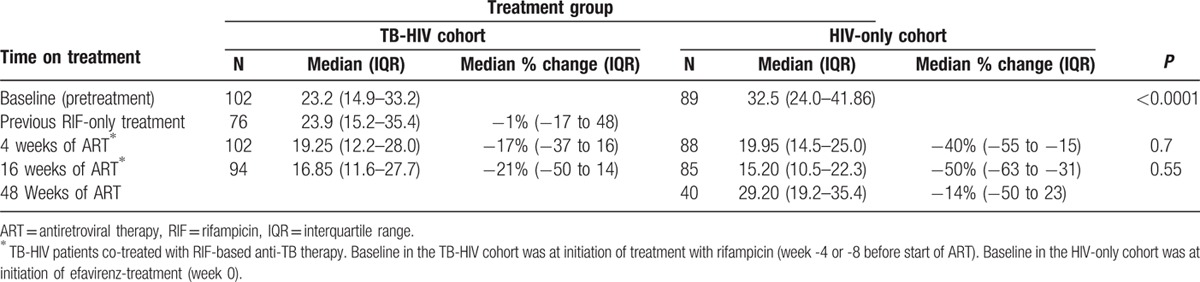
Comparison of median and IQR of plasma 25 (OH)D_3_ (nmol/L) levels at baseline and during treatment.

**Figure 2:**
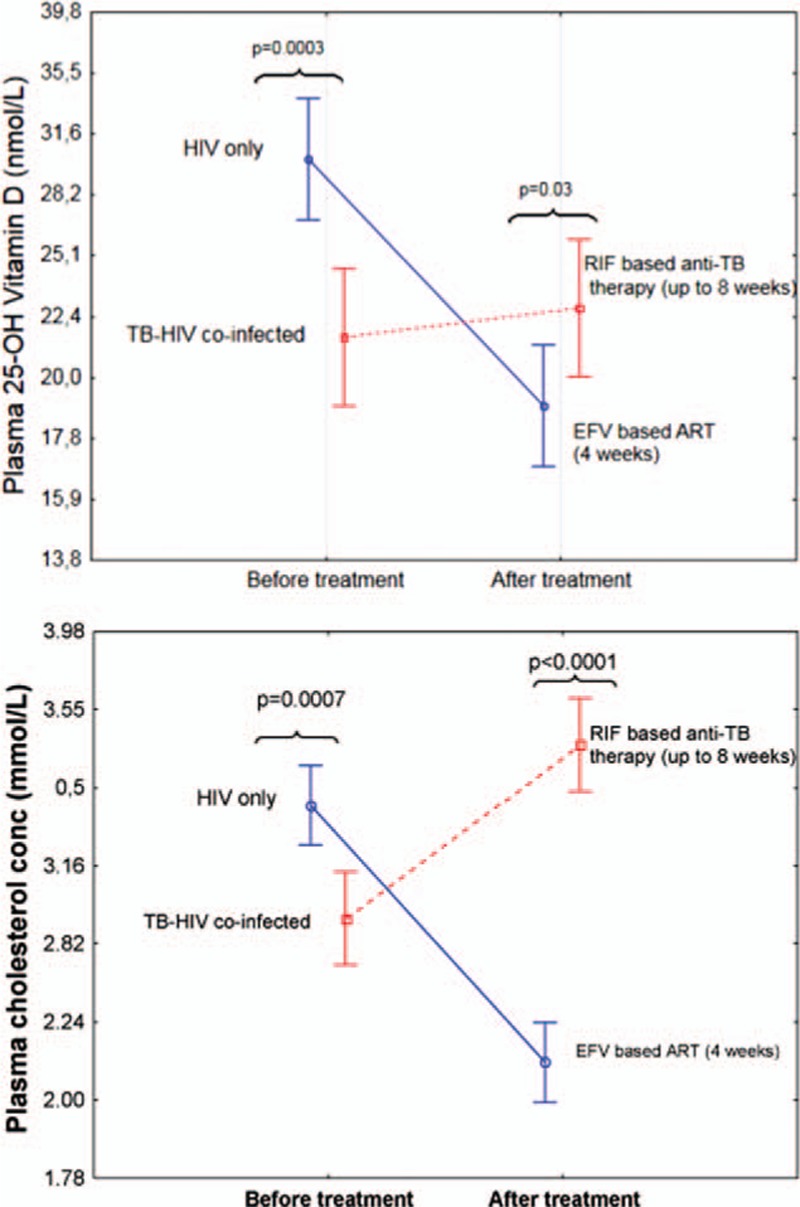
Change in the mean (log) plasma 25 (OH)D_3_ (upper panel) and cholesterol levels (lower panel) from baseline by 4 weeks of efavirenz-based cART in HIV-only patients (straight line) versus up to 8 weeks of rifampicin-based anti-tuberculosis (TB) therapy only in TB-HIV co-infected patients (broken line). Between-treatment group differences were analyzed using unpaired *t* test. Vertical bars denote 0.95 confidence intervals of the mean.

Comparisons of change in log plasma 25 (OH)D_3_ levels from baseline after 4 weeks of efavirenz-based cART alone versus 4 or 8 weeks of rifampicin-based anti-TB therapy alone are presented in Figure 2. TB-HIV co-infected patients had significantly lower mean log plasma 25 (OH)D_3_ levels compared to HIV-only patients at baseline (*P* < 0.001, Fig. 2), which became reversed soon after initiating therapy. The mean log plasma 25 (OH)D_3_ levels became significantly lower in HIV-only patients after 4 weeks of efavirenz-based cART compared to 4 to 8 weeks of rifampicin-based anti-TB treatment only in TB-HIV patients (*P* = 0.03). Initiation of cART in TB-HIV patients on anti-TB therapy lowered the mean plasma 25 (OH)D_3_ levels, and there was no significant difference in the mean plasma 25 (OH)D_3_ levels between the 2 treatment groups at weeks 4 and 16 of cART.

**Figure 3:**
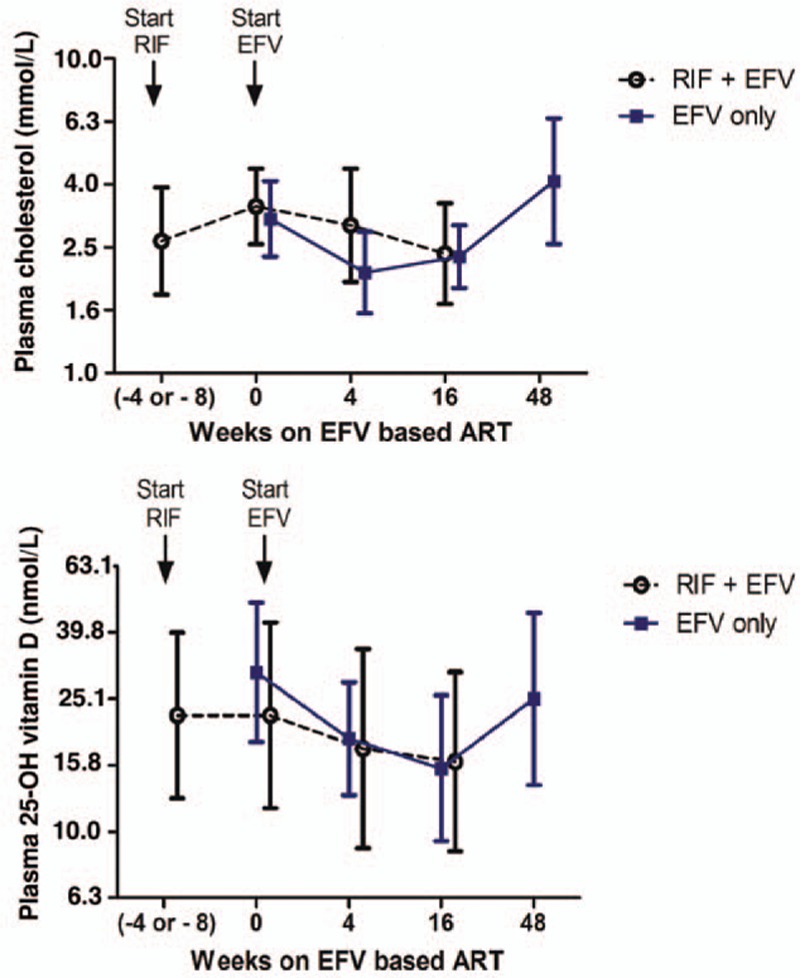
Change in the mean (log) plasma levels of cholesterol (upper panel) and 25 (OH)D_3_ (lower panel) from baseline during efavirenz-based cART alone in HIV patients (EFV only, straight line) or with rifampicin-based anti-tuberculosis therapy in TB-HIV co-infected patients (RIF + EFV, broken line). Between-treatment group differences were analyzed using unpaired *t* test. Vertical bars denote 0.95 confidence intervals of the mean.

The overall change in the mean log plasma 25 (OH)D_3_ levels profile from baseline during efavirenz-based cART alone versus rifampicin-based anti-TB co-treatment is presented in Figure 3. In HIV-only patients, within-treatment group analysis over time (using repeated measure ANOVA) indicated that efavirenz-based cART significantly reduced the mean log plasma levels of 25 (OH)D_3_ (*P* < 0.001). Paired *t* tests indicated significantly lower plasma 25 (OH)D_3_ levels at week 4 (*P* < 0.001, geometric mean ratio [GMR] = 1.606; 95% confidence interval [CI] of the mean GMR = 1.46–1.76) and at week 16 (*P* < 0.001, GMR = 1.99; 95% CI of GMR = 1.76–2.21) compared with the baseline pretreatment value. As cART continued, the plasma 25 (OH)D_3_ levels were restored, and no significant difference from baseline was found after 48 weeks of cART (*P* = 0.12) in HIV-only patients.

In TB-HIV co-infected patients, within-treatment group analysis indicated that efavirenz-based cART significantly reduced mean log plasma levels of 25 (OH)D_3_ (*P* < 0.0001). Paired *t* tests indicated significantly lower 25 (OH)D_3_ level at week 4 (*P* = 0.01, GMR = 1.17; 95% CI of GMR = 1.04–1.34) and week 16 (*P* < 0.001, GMR = 1.41, 95% CI of GMR = 1.18–1.69) compared to the baseline value. Data on plasma 25 (OH)D_3_ at week 48 of cART in TB-HIV co-infected patients were not available.

### 3.2 Vitamin D deficiency at baseline and during treatment

All patients had deficient (92%) or insufficient (8%) plasma 25 (OH)D_3_ levels at the study enrollment. Despite receiving cART and anti-TB therapy, none of the patients achieved a sufficient (>72.5 nmol/L) plasma 25 (OH)D_3_ level during the study follow-up period.

The prevalence of SVDD before starting treatment (at baseline) and at the 4^th^, 16^th^, and 48^th^ weeks of efavirenz-based cART in each treatment group is presented in Table 3. At baseline, 57% (95% CI = 48.3%–67.5%) of TB-HIV co-infected patients had SVDD, whereas only 27% (95% CI = 15.1%–32.9%) of HIV-only infected patients had SVDD (*P* < 0.001). In the HIV-only cohort, initiation of efavirenz-based cART significantly increased the proportion of patients with SVDD from 27% at baseline to 76% (95% CI = 67.1%–84.5%) at week 4, and 79% (95% CI = 70.3%–87.3%) at week 16, and 42.5% (95% CI = 32.2%–52.7%) at week 48 cART. Whereas in TB-HIV patients on rifampicin-based anti-TB therapy, initiation of efavirenz-based cART co-treatment increased the proportion of patients with SVDD from 57% at baseline to 70% (95% CI = 69.7%–78.5%) at week 4 and 72% (95% CI = 63.6%–80.9%) at week 16 of cART.

**Table 3:**
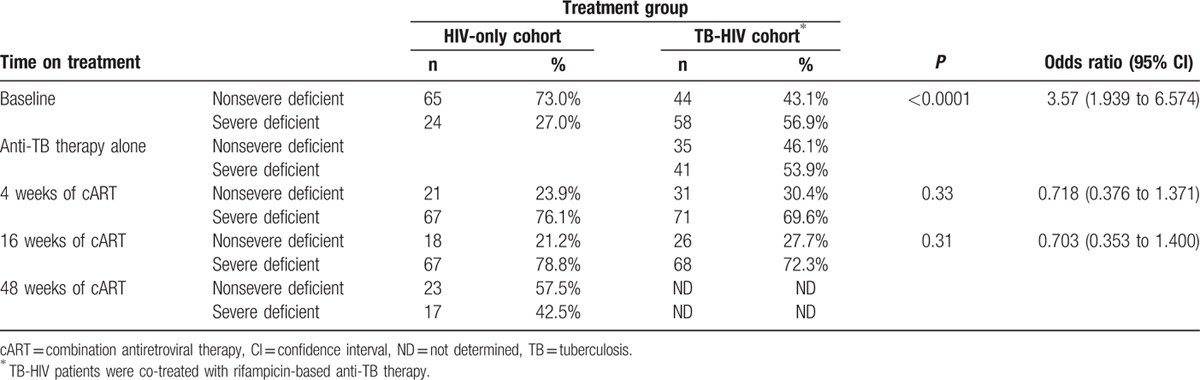
Comparison of proportions of patients with severe vitamin D deficiency (defined as plasma 25 (OH)D_3_ <25nmol/L) between treatment groups: HIV patients treated with efavirenz-based cART alone (HIV-only cohort) versus TB-HIV co-infected patients co-treated with efavirenz-based cART and rifampicin-based anti-TB therapy (TB-HIV cohort).

### 3.3 Plasma cholesterol levels at baseline and during treatment

The pretreatment mean log plasma cholesterol level was significantly higher in HIV patients compared to TB-HIV patients. Initiation of cART in HIV-only patients significantly reduced the plasma level of cholesterol. In contrast, increased plasma levels of cholesterol after 4 to 8 weeks of rifampicin-based anti-TB therapy alone were observed in TB-HIV patients (Fig. 2). The median percent reduction from baseline in cholesterol level by 4 weeks of efavirenz-based cART was −33% and the respective increase by rifampicin-based anti-TB therapy treatment alone was 21%. There was a similar pattern of change in plasma cholesterol and 25 (OH)D_3_ level particularly in HIV-only patients treated with efavirenz-based cART (Fig. 3).

### 3.4 Efavirenz plasma concentrations

The median (IQR) of efavirenz plasma concentrations in HIV-only and TB-HIV patients at week 4 of cART was 1200 (841–1846) ng/mL and 1574 (984–2041) ng/mL, respectively (*P* = 0.45). The median and IQR of efavirenz plasma concentrations in HIV-only and TB-HIV patients at week 16 of cART were 1328 (980–1947) ng/mL and 1236 (826–2013) ng/mL, respectively (*P* = 0.26).

### 3.5 Predictors of low vitamin D status at baseline and during treatment

Linear, regression analysis was used to identify predictors of low plasma 25 (OH)D_3_ at baseline (Table 4). In a univariate analysis, low plasma 25 (OH)D_3_ levels at baseline were significantly associated with TB-co-infection, low Karnofsky score, low body mass index, low plasma cholesterol level, low hemoglobin level, low albumin, high viral load, high CYP3A activity (as measured by 4β-hydroxycholesterol to cholesterol ratio), and high serum alkaline phosphatase. In a stepwise multivariate regression analysis, low Karnofsky score, low albumin level, high viral load, and high 4β-hydroxycholesterol to cholesterol ratio remained significant predictors of low 25 (OH)D_3_ concentration at baseline.

**Table 4:**
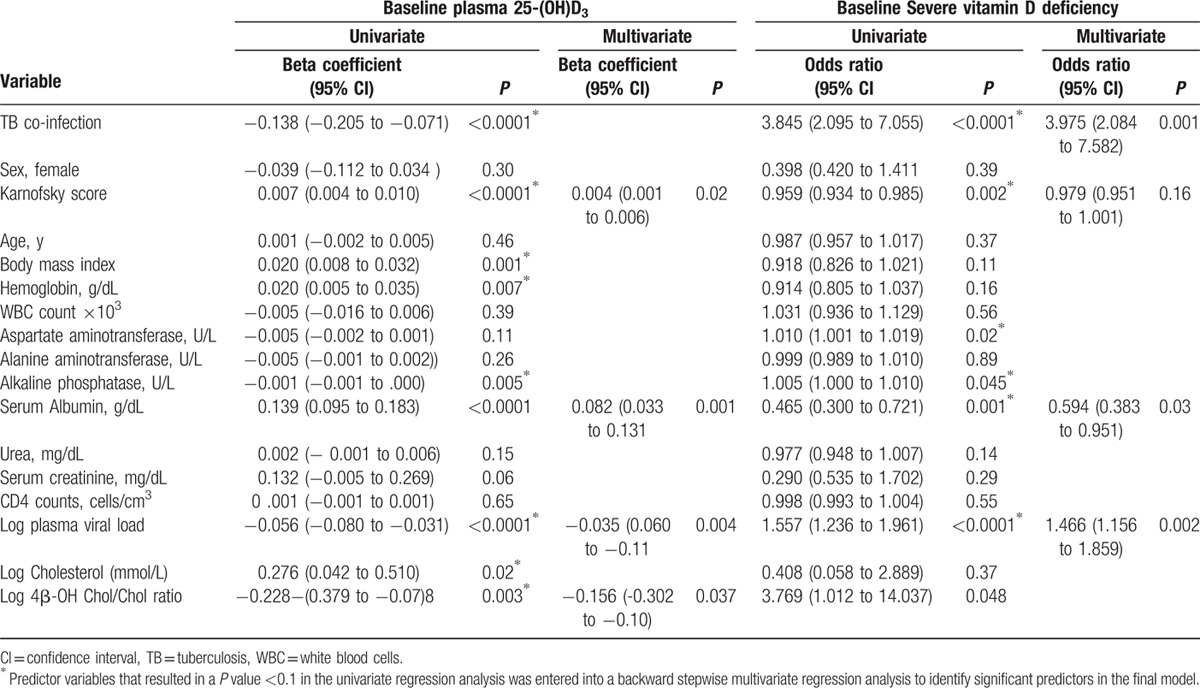
Predictors of pre-treatment plasma 25 (OH)D_3_ level and severe vitamin D deficiency (25 (OH)D3 <25 nmol/L) using linear regression and logistic regression analysis respectively.

Univariate logistic regression identified TB co-infection, low Karnofsky score, high aspartate aminotransferase, low albumin levels, high 4β-hydroxycholesterol to cholesterol ratio, and high HIV viral load as significant predictors of SVDD at baseline. Multivariate logistic regression, using a backward stepwise conditional model of all variables with a *P* value <0.1 in the univariate analysis (Table 4), identified TB co-infection, high HIV viral load, and low albumin level as significant predictors of SVDD at baseline.

After 4 weeks of efavirenz-based cART, low Karnofsky score and albumin level at baseline, low plasma cholesterol, high efavirenz plasma concentration, and high 4β-hydroxycholesterol to cholesterol ratio at week 4 of cART were significant predictors of a low 25 (OH)D level soon after efavirenz-based cART initiation (Table 5). In a multivariate regression analysis, low Karnofsky score at baseline, high-current efavirenz plasma concentration, and high-current 4β-hydroxycholesterol to cholesterol ratio remained significant predictors of a low 25 (OH)D level at week 4 of cART (Table 5).

**Table 5:**
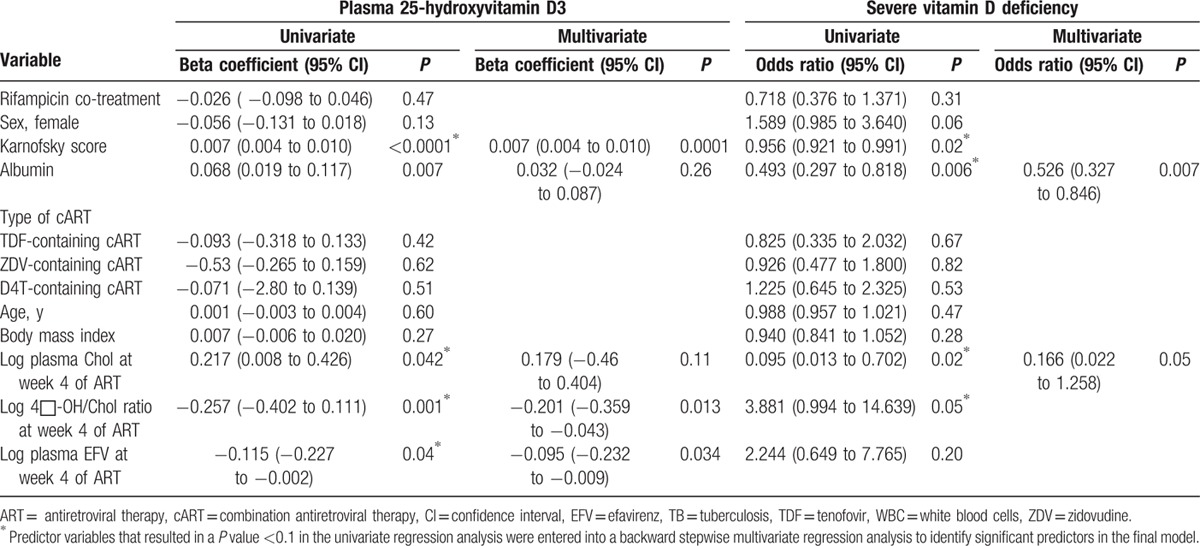
Predictors of plasma 25 (OH)D_3_ level and severe vitamin D deficiency (25 (OH)D3 <25 nmol/L) at 4 weeks of EFV-based cART using simple linear regression and logistic regression respectively.

In a univariate logistic regression analysis, low albumin at baseline, low Karnofsky score at baseline, low cholesterol, and high 4β-hydroxycholesterol to cholesterol ratio were significant predictors SVDD at week 4 of cART (Table 5). In a multivariate analysis, low albumin at baseline and low-current plasma cholesterol level remained a significant predictor of efavirenz-induced SVDD at week 4 of cART. None of the predicting variables tested were significant predictors of low 25 (OH)D_3_ levels at week 16 of cART.

## 4 Discussion

We performed a prospective observational study to identify predictors of a low 25 (OH)D_3_ level before starting therapy and during efavirenz-based cART, anti-TB or a combination thereof in treatment-naïve HIV patients with or without TB co-infection. The main finding of this study includes: a significant association of a low plasma 25 (OH)D_3_ level at baseline with TB-co-infection and HIV disease progression; efavirenz-based cART significantly reduces plasma 25 (OH)D_3_ levels and this effect is more pronounced when given alone than with rifampicin-based anti-TB therapy; low plasma cholesterol level, high CYP3A activity, and high plasma efavirenz concentration are predictors of early cART-induced low 25 (OH)D_3_ level.

Seasonal variation affects vitamin D status, particularly in countries far from the equator, where the lower angle of the sun and more cloud cover during the winter result in less UV-B exposure and hence low production of vitamin D in skin.^[30,31]^ However, lack of sunshine-derived vitamin D deficiency is not expected in countries around the equator.^[32,33]^ Ethiopia is located in east Africa close to the equator (3 degree N to 14.8 degree latitude) and there is abundant sunshine to form vitamin D all year round.^[34]^ This study was conducted in Addis Ababa, the capital city of Ethiopia, located at latitude 9 degree N. In the present study, we found no significant influence of seasonal variation (rainy versus dry seasons) on plasma 25 (OH)D_3_ concentrations. Our finding is consistent with previous findings from countries located near the equator.^[32,33]^

Despite the all-year-round sunshine providing abundant UVB radiation, high prevalence of vitamin D deficiency (42%) and insufficiency (49%) in Ethiopian healthy adolescents and children was reported previously.^[35,36]^ HIV patients and patients with active TB have lower levels of 25 (OH)D_3_ than healthy controls and patients with latent TB.^[37,38]^ Accordingly, all patients who participated in this study had deficient (92%) or insufficient (8%) plasma 25 (OH)D_3_ levels at study enrollment, and none of them achieved a sufficient plasma 25 (OH)D_3_ level (>72.5 nmol/L) during a 1-year cART follow-up period. The prevalence of vitamin D deficiency in our treatment-naive HIV-only and TB-HIV patients from Ethiopia is quite high compared to other reports from HIV patients in East Africa. Low prevalence of vitamin D insufficiency at baseline in TB-HIV co-infected (41%) and HIV (35%)-only infected patients from Uganda has been reported.^[39]^ Likewise, only 9.2% and 43.6% of HIV patients from Tanzania were vitamin D-deficient and -insufficient, respectively.^[40]^ The finding of a high prevalence of SVDD in Ethiopian HIV patients might be because our study population consisted of very ill patients with low CD4 cell count (<200 cells/mm^3^) at baseline and a majority presented HIV stage 3 or 4 indicating progression to AIDS (Table 1). Association of nadir CD4 cell count <200 cells/mm^3^ and HIV/TB disease progression with SVDD was reported previously.^[25,37]^

In our regression analysis (Table 4), low plasma 25 (OH)D_3_ and SVDD at baseline was significantly associated with high viral load, which is a marker for HIV disease progression.^[41]^ Our finding is in line with previous reports from the literature describing association of vitamin D deficiency with HIV disease progression.^[42]^ Variations in the dietary source containing vitamin D may also contribute to between-population differences in vitamin D status. TB co-infected HIV patients had significantly lower levels of 25 (OH)D_3_ than patients without TB co-infection. Our finding further indicates that severe vitamin D deficiency in HIV patients is a risk factor for the development of active TB.^[40,43]^

Treatment with rifampicin-based anti-TB treatment (up to 8 weeks) before the start of cART in TB-HIV co-infected patients resulted in no significant change in 25 (OH)D_3_ levels from baseline. However, a significant reduction of plasma 25 (OH)D3 levels was noted soon after initiating cART in both HIV-only and TB-HIV co-infected patients treated. The effect of efavirenz-based cART in lowering 25 (OH)D_3_ levels was more pronounced when given alone than when given together with rifampicin-based anti-TB therapy. In HIV only cohort, initiation of efavirenz-based cART increased the proportion of patients with SVDD by 3-fold at week 4 of cART, which persisted until week 16. As therapy continued, vitamin D levels were gradually restored, but 42% of the patients still had SVDD after 48 weeks of cART (Table 3). In contrast, in TB-HIV patients on anti-TB co-treatment initiation of efavirenz-based cART increased the proportion of patients with SVDD from 57% at baseline to 70% and 72% at 4 and 16 weeks of cART, respectively.

Previous in vitro studies suggested that a low 25 (OH)D_3_ level may be related to an increased catabolism of 25 (OH)D through CYP3A4 enzyme induction.^[18]^ In line with this, we found a significant correlation between CYP3A enzyme activity, as measured by 4β-hydroxycholesterol to cholesterol ratio, and low 25 (OH)D concentration both at baseline and week 4 of cART. But as therapy continued, no significant association between CYP3A activity and vitamin D status was observed. We previously reported a significant association between plasma efavirenz concentration and 4β-hydroxycholesterol/cholesterol ratio in HIV patients.^[10]^ In the present study, we found a significant correlation between plasma efavirenz concentration and 25 (OH)D_3_ concentration. Vitamin D is involved in the regulation of CYP3A enzyme expression.^[18,19]^ CYP3A4 enzyme activity and expression are induced by 1,25-(OH)D_3_ via the vitamin D receptor.^[44]^ In addition, CYP3A catalyzes the 4-hydroxylation of 25 (OH)D_3_ and hence CYP3A induction may contribute to drug-induced vitamin D deficiency.^[18,19]^

Rifampicin is a more potent inducer of CYP3A than efavirenz.^[14]^ Thus, it is plausible to expect a more pronounced effect of rifampicin in lowering the 25 (OH)D_3_ level compared to efavirenz-based cART alone. However, we found no significant effect of rifampicin-based anti-TB therapy (given before the initiation of cART), on the vitamin D status (Table 3). Based on our finding, the contribution of CYP3A enzyme induction by rifampicin per se may not be the major underlying mechanism, and perhaps other mechanism such as cholesterol-increasing effect of rifampicin may play a role to counterbalance the effect of efavirenz in modulating vitamin D status as discussed below. We found no significant correlations between CYP3A activity and vitamin D status at 16 and 48 weeks of cART. Probably, the importance of CYP3A catalyzed vitamin D metabolism may diminish, with the increased wellbeing of the patient during long-term cART. Indeed, improved CD4 recovery and vitamin D repletion in HIV patients on cART were reported recently. Consistent with our finding, 25 (OH)D levels have recently been shown to decline up to 24 weeks following initiation of efavirenz-based cART, but to stabilize thereafter.^[45]^

Previously, we reported that initiation of efavirenz-based cART in HIV patients significantly lowers the plasma cholesterol level transiently.^[10]^ 7-dehydrocholesterol is a precursor to both cholesterol and vitamin D. Thus, efavirenz-based cART lowers the vitamin D level possibly by modulating the precursor concentration. To evaluate this, we monitored the change in plasma 25 (OH)D_3_ alongside the corresponding plasma cholesterol level in each patient before starting therapy and at different time points during treatment. Notably, TB-HIV co-infected patients had significantly lower pretreatment plasma concentration of both cholesterol and vitamin D compared to HIV-only patients. A similar pattern of change in plasma cholesterol level and vitamin D status over time was observed during cART, particularly when given alone (Fig. 3). Interestingly, initiation of efavirenz treatment significantly lowered both the plasma cholesterol and 25 (OH)D_3_ level dramatically in both treatment groups, but this effect was more pronounced when efavirenz-based cART was given alone than with rifampicin-based anti-TB co-treatment. Indeed, there was a significant positive correlation between plasma cholesterol level and vitamin D status during cART, particularly at week 4. Though not significant, a similar trend was observed at 16 and 48 weeks of cART. This may indicate that reduction in 25 (OH)D_3_ concentration by early initiation of efavirenz might be secondary to change in cholesterol concentration. In line with this, a recent in vitro study reported that treatment of cells with cholesterol resulted in a 3-fold increase in vitamin D relative to cholesterol synthesis, demonstrating that cholesterol feeds back via 7-dehydrocholesterol reductase (DHCR7) increasing vitamin D production.^[46]^ Indeed our results indicate that rifampicin-based anti-TB therapy alone significantly increased the plasma cholesterol level (Fig. 2). Thus, rifampicin-based anti-TB treatment might counterbalance the vitamin D-lowering effect of efavirenz to some extent by increasing the plasma cholesterol level and hence the vitamin D level in TB-HIV patients co-treated with rifampicin.

Increased plasma levels of 25 (OH)D_3_ after 1 year of cART has been reported previously in Japanese male HIV patients.^[47]^ Similarly, our study shows that at the 48^th^ week of cART in HIV-only patients, both the plasma levels of 25 (OH)D_3_ and cholesterol levels restored gradually, which may be because of overall improved health status. In diverse HIV-infected populations, Vitamin D insufficiency and deficiency are associated with HIV disease progression, and virological failure after antiretroviral therapy initiation.^[45]^ Recent randomized clinical trials indicated that vitamin D supplementation improved CD4 recovery and vitamin D repletion suggesting potential benefit on immunologic recovery during cART.^[48,49]^

### 4.1 Study Limitations

This study has some limitations. First, the study participants consisted of patients who had CD4 cell count <200 at baseline, following the WHO and Ethiopian national HIV treatment guideline valid during the study period. Thus, the result may not be directly extrapolatable to patients with high CD4 cell count at baseline. Second, since vitamin D status is influenced by genetic variations, darker skin, exposure to the sunshine, and dietary sources, the study result from a single study location and Ethiopian population may not be applicable for genetically diverse non-black populations living in the nontropical zone.

## 5 Conclusions

The finding of this study suggests TB incidence and HIV disease progression are associated with low levels of 25 (OH)D_3_ at baseline. Early initiation of efavirenz-based cART is associated with low plasma 25 (OH)D_3_ levels and high incidence of SVDD, which sustains up to 16^th^ week of cART. This effect is most pronounced when given alone than with rifampicin-based anti-TB co-treatment. As cART continues, 25 (OH)D_3_ levels restore gradually with time. Rifampicin-based anti-TB treatment initiated before cART has no significant effect on the 25 (OH)D_3_ levels. High plasma efavirenz concentration, high CYP3A activity, and low plasma cholesterol are predictors of early cART-induced low plasma 25 (OH)D_3_ level. Considering the very low levels of 25 (OH)D_3_ in both patient groups, supplementary vitamin D may be beneficial not only for TB-HIV patients, but also for HIV-only patients at the initiation of cART and anti-TB treatment.
